# Health service use and costs among migrants in an irregular situation: Cross-sectional register-based study from a voluntary-based clinic

**DOI:** 10.1093/pubmed/fdab382

**Published:** 2021-10-30

**Authors:** Nina Tjukanov, Paula Tiittala, Heli Salmi

**Affiliations:** Global Clinic Helsinki; University of Vaasa, Department of Social and Health Management, 65200 Vaasa, Finland; Global Clinic Helsinki; Global Clinic Helsinki; Pediatric Research Center, University of Helsinki and Helsinki University Hospital, 00029 Helsinki, Finland

**Keywords:** entitlements to healthcare, healthcare, healthcare costs, immigrant, undocumented

## Abstract

**Background:**

As few data based on actual demand for healthcare services in vulnerable migrant populations exist, we studied service use and healthcare costs in a cohort of migrants in an irregular situation.

**Methods:**

In this single-centre retrospective register study, we examined the reasons for encounter, diagnoses, service use and costs of healthcare among patients at a voluntary clinic for migrants in an irregular situation in Helsinki, Finland. ICPC-2 classification and national unit costs for primary healthcare were used for the cost estimation.

**Results:**

A total of 546 patient visits accounted for 620 ICPC-2 coded reasons for encounter, diagnoses and process codes. The most common health problems were teeth/gum disease (10%), acute upper respiratory infection (5%) and oesophageal disease (3%). Visits seldom led to complementary investigations (2%), follow-up visits (5%) or referrals (11%). The total cost of treatment, excluding dental health costs, was 71 euros per visit.

**Conclusions:**

Migrants in an irregular situation present with a variety of health concerns, the majority of which can be treated in a basic primary healthcare facility at a relatively low cost. This encourages research to evaluate the health and cost effects of extending public healthcare for migrants in an irregular situation beyond emergency care.

## Background

European countries have different healthcare policies for irregular immigrants. Most European countries offer emergency care to everyone^1^ but payment policies differ.^2^ Some countries recognize the need to provide broader services for vulnerable populations such as children.^1^

Finland is a high-income country with public health insurance based on residency. In our healthcare system, any immigrant without an official immigration status granting entitlement to publicly financed healthcare is considered an irregular immigrant. Migrants in an irregular situation, including children and pregnant women, are entitled to urgent care at their own cost. Preventive care, medication or follow-up for chronic conditions are not provided. The number of migrants in an irregular situation in Finland is estimated at 2000–10 000, with the majority residing in Helsinki capital area. Since 2013, the city of Helsinki has granted access to healthcare beyond emergency care for children and pregnant women in an irregular situation.

Medical practitioners widely agree on the ethical, medical and humanitarian justification and cost-effectiveness of providing necessary healthcare for all.^3–5^ Proposals on broader entitlements to healthcare services for migrants raise concerns about costs and burden to the public healthcare system. However, few data based on actual healthcare needs and costs exist to support decisions.

Thus, we explored health problems, service use and costs of non-urgent healthcare among patients at a voluntary clinic (Global Clinic) for migrants in an irregular situation in Helsinki, Finland. Global Clinic is a free-of-charge, weekly walk-in clinic run by volunteers, offering anonymous primary healthcare services for migrants in an irregular situation. The multiprofessional team consists of health professionals, lawyers and interpreters.

## Methods

All patient visits to health professionals in Global Clinic from 1 January to 31 December 2016 were included. Basic sociodemographic characteristics, reasons for encounter, re-encounters, diagnoses, treatment and referral were collected from the electronic health record (ASTA ®). Visits with missing information were excluded (*N* = 3).

Reasons for encounter and diagnoses were converted to International classification of primary healthcare codes (ICPC-2).^6^ Causal codes were preferred over symptom codes and other codes over process and operation codes.

Healthcare costs were calculated based on ICPC-2 codes using unit costs of primary healthcare services in Finland for 2011^7^ and adjusted to inflation.^8^ Dental problems and operation codes were excluded from the cost analysis, as their costs were not available.

The ethics committee of Helsinki Deaconess Institute approved the study in 2013 and 2016.

## Statistical analyses

Descriptive statistics were calculated with MS Excel 2016. IBM SPSS Statistics 25 was used to compare means with independent samples t-test and categorical variables with Pearson chi squared. *P* values <0.05 were considered statistically significant.

## Results

Altogether 556 patient visits were included (Table 1). The mean age was 35 years (range 0–69) without statistically significant difference by gender (*p* = 0.10). Children represented 4% of all visits. Three-fourths of visits represented patients from other EU countries.

**Table 1:** Basic sociodemographic characteristics of patient visits in the clinic 2016; n(%). Total *n* = 556

Age	n (%)
0–6	5 (1)
7–16	18 (3)
17–29	168 (30)
30–45	212 (38)
46–65	105 (19)
66-	2 (0)
Age unknown	46 (8)
**Sex**	**n (%)**
Female	235 (42)
Male	306 (55)
Sex unknown	15 (3)
**Region of origin**	**n (%)**
Europe	408 (73)
North-Africa and the Middle East	28 (5)
Sub-Saharan Africa	81 (15)
Asia	15 (3)
America	4 (1)
Region of origin unknown	20 (4)

Follow-up visits represented 37% (*n* = 203) of all visits. Two percent of visits resulted in complementary investigations, 5% in follow-up visits to the clinic and 11% in a referral to public healthcare, most often to an emergency department (80%) and to maternal and child health centres (18%).

Altogether 620 ICPC-2 codes were registered to 546 visits. A total of 10 visits did not receive any ICPC-2 code. Twelve percent (n = 64) of all visits had more than one ICPC-2 code.

**Fig. 1:**
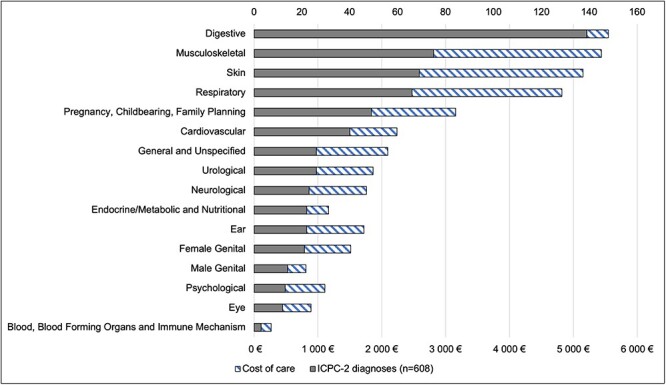
ICPC-2 codes (*n* = 620) and costs categorized to ICPC-2 (International Classification of Primary Care) chapters.

The most common health concerns belonged to digestive (22%), musculoskeletal (12%) or dermatological (11%) ICPC-2 categories (Fig. 1). The three most common ICPC-2 diagnoses were teeth/gum disease (10%) included in the digestive category, acute upper respiratory infection (5%) and oesophageal disease (3%). No statistically significant differences by gender were observed among these most common health concerns. For women, 17% of visits were related to pregnancy, childbearing or family planning.

The total cost of care was 39 547 euros, or 71 euros per visit on average. Digestive, musculoskeletal and dermatological problems represented 41% of the total costs (Fig. 1). For women, 10% of costs were related to antenatal follow-up.

## Discussion

### Main finding of this study

Migrants in an irregular situation had medical complaints covering the whole range of medical specialties. Still, most health problems were amenable to treatment in a very basic healthcare setting. Accordingly, the cost of the treatment would have been relatively low, if the treatment had been provided in public primary healthcare.

### What is already known on this topic

Our results are in line with previous studies from European countries reporting varying healthcare needs,^9^ low incidence of tropical diseases, tuberculosis or HIV,^9^^,^^10^ frequent obstetric or gynaecological problems^9^ and very few psychiatric conditions.^11^

### What this study adds

The average cost per visit in Global Clinic was lower than for a general practitioner’s encounter in Finland.^7^ According to estimates^4^ and actual service use^12^^,^^13^ and costs,^14^ extending migrants’ healthcare beyond emergency care is likely to cost less than estimated based on costs in the general population.

Immigrants in an irregular situation use less healthcare services than they are entitled to.^13^^,^^15^^,^^16^ Multiple administrative, economic, language and cultural barriers, and fear of authorities, decrease the accessibility of services. Accordingly, despite women being entitled to public free-of-charge maternity care, pregnancy was a common reason for encounter at our voluntary clinic. Thus, access to prenatal care can be encouraged by establishing low-threshold services in relevant languages, and trust.

The low number of children at our clinic is similar to that reported from Denmark.^9^ It can reflect the age distribution of irregular migrant population in Nordic countries, or lower barriers to public healthcare compared to adults.

### Limitations of this study

As the demographics of migrants in an irregular situation in the study area are unknown, we were unable to compare the reasons for encounter and service use with the general population.

We conclude that the costs of primary-level non-emergency healthcare to migrants in an irregular situation are low, and the complaints can often be treated with simple means. More research is needed to understand the health and cost benefits of extending public healthcare services for migrants in an irregular situation beyond emergency care. In addition, barriers leading to suboptimal use of existing services should be identified by interviewing migrants in an irregular situation and their healthcare providers.

## Notes

**Nina Tjukanov**, RM MPA

**Paula Tiittala**, MD PhD

**Heli Salmi**, MD PhD
